# Effect of Physical Exercise on College Students’ Life Satisfaction: Mediating Role of Competence and Relatedness Needs

**DOI:** 10.3389/fpsyg.2022.930253

**Published:** 2022-07-29

**Authors:** Yunqi Zhang, Menghao Ren, Shengqi Zou

**Affiliations:** ^1^Faculty of Education, Southwest University, Chongqing, China; ^2^School of Education and Psychology, Southwest Minzu University, Chengdu, China; ^3^Department of Psychology, Center for Mind and Brain Science, Cognition and Human Behavior Key Laboratory of Hunan Province, Hunan Normal University, Changsha, China

**Keywords:** physical exercise, competence need satisfaction, relatedness need satisfaction, life satisfaction, college students

## Abstract

This study examined the effect of physical exercise on the life satisfaction among college students. On the basis of the Basic Psychological Need Theory, the mediating roles of competence and relatedness needs satisfaction and their differences among college students in physical education (PE) majors and non-PE majors were explored. The sample included 1,012 college students who were selected to participate in an online survey. Major findings were as follows: (1) The total effect of physical exercise commitment on college students’ life satisfaction was marginally significant while that of physical exercise adherence was not significant; (2) The effect of physical exercise commitment was observed exclusively through the mediating role of relatedness need satisfaction, while that of physical exercise adherence was through both competence and relatedness needs satisfaction; (3) In terms of differences caused by major, only one mediation path, that was, physical exercise → competence need satisfaction → college students’s life satisfaction was significant among PE majors. This study thus enriched the empirical research on the benefits of physical exercise to individual mental health, highlighted the particularity of college students majoring in PE, and provided targeted and sensible suggestions for the design of physical exercise intervention programs.

## Introduction

The benefits of physical exercise have long been of concern to researchers and practitioners. Physical exercise is widely proven to not only reduce the risk of cardiovascular and cerebrovascular diseases, diabetes, hypertension, and even cancer, but also alleviate various psychological and behavioral problems (Curran et al., 2020). A series of meta-analyses have found that physical exercise can considerably alleviate depression and anxiety among HIV-infected patients (Heissel et al., 2019), relieve inattention—the major symptom of ADHD in children (Cerrillo-Urbina et al., 2015), and effectively improve the sleep quality of patients with post-traumatic stress disorder (McGranahan and O’Connor, 2021). However, literature has mainly focused on the effect of physical exercise on ameliorating psychological and behavioral problems in special groups; less attention has been paid to the general population and positive psychological adaption, which is an integral part of identifying the benefits of physical exercise.

Physical exercise is an inclusive concept that can be analyzed from multiple perspectives. Empirical studies have focused on the effects of the intensity, duration, and form of physical exercise on individual physical and mental health. For example, moderate to vigorous physical activities were found to considerably improve the physical fitness and performance of diabetic patients (Taylor et al., 2014); individuals who exercised more, especially those who spend 30–60 mins, scored higher on physical health, mental health, and academic performance (Shantakumar et al., 2022); the combination of aerobic exercise and yoga could effectively reduce depression in individuals (Alderman et al., 2016). However, other characteristics of physical exercise, such as commitment and adherence, have received less attention in research that examines the positive role of physical exercise in mental health.

Physical exercise commitment refers to a psychological state of an individual’s desire and determination to continue carrying out such activities (Martin and Hausenblaus, 1998) while adherence refers to an individual’s behavioral tendency to maintain a pre-established exercise schedule for a specific period of time (Chapman et al., 2015). In recent years, the influencing factors of physical exercise commitment and adherence have been extensively examined from different perspectives to increase participation in physical intervention programs and indirectly promote physical and mental health (Kang et al., 2020; Evans et al., 2022). However, whether and how physical exercise commitment and adherence affect individual mental health, including life satisfaction (Jiang et al., 2018; Sullins, 2019). Therefore, this study aimed to examine the effect and mechanism of physical exercise commitment and adherence on college students’ life satisfaction and provided new supporting evidence to deepen the understanding regarding its benefits.

Numerous theoretical viewpoints have explained the decrease in negative psychological reactions, such as depression and anxiety, and the increase in psychological adaption brought about by physical exercise (Arent et al., 2020). Among these views, those that emphasize the mediating roles of exercisers’ psychological factors have received extensive attention. First, during physical exercise, exercisers gradually build a stronger sense of control over themselves and the environment, which helps to alleviate depression and anxiety as well as increase psychological resilience (Landers and Arent, 2007). In addition, physical exercise is not only a major form of social interaction but also a vital way to gain social support. Thus, physical exercise can improve individuals’ interpersonal skills and the quality of interpersonal relationships, thereby enhancing their psychological adaption (Gairns et al., 2015).

In fact, from the perspective of Basic Psychological Need Theory, the above viewpoints highlighted the mediating role of the satisfaction of individual competence and relatedness needs (Rodriguez, 2020). Competence need refers to individuals’ need to feel confident in their own abilities to efficiently and proficiently attain specific goals, while relatedness need refers to individuals’ desire to connect with others and be respected and valued (Deci and Ryan, 2000). Through active participation in physical exercise, individuals’ competence and self-efficacy can significantly improve (Xu et al., 2021). Empirical studies have also found that physical exercise can positively predict individuals’ competence need (Babic et al., 2014), the satisfaction of which can promote their positive expectations of behavioral outcomes (Behzadnia and Fatahmodares, 2020). As such, they can be full of hope for the present and the future, that is, be more satisfied with life. Similarly, physical exercise is also an important medium for interpersonal communication, which can significantly increase the sense of belonging and help to satisfy the relatedness need (Fraguela-Vale et al., 2020), thereby enhancing an individuals’ courage and confidence in the face of pressure, setbacks, and difficulties, and ensuring their subjective well-being. Therefore, this study aimed to explore whether the satisfaction of competence and relatedness needs has mediating roles in the relationship between physical exercise commitment and adherence and college students’ life satisfaction.

Notably, despite the generally recognized effect of physical exercise on mental health, the magnitude and mechanism of this effect vary from individual to individual (Landers and Arent, 2007). When examining the relationship between physical exercise and psychological problems such as depression and anxiety, literature has focused on comparing the differences between clinical and non-clinical samples, finding that physical exercise is more effective for patients with depression or anxiety than for healthy people (Arent et al., 2020). For college students, their majors are also individual characteristics that need investigation. The core objective of physical education (PE) majors is the cultivation of sports skills and the development of an exercise habit, which has resulted in their better performance on physical exercise commitment and adherence as compared with non-PE majors. In addition, empirically, college students in PE majors have a stronger commitment to physical exercise, and even under extreme conditions such as illness and injury, are more inclined to carry out such activities and even develop a lifelong habit of exercising, indicating a stronger physical exercise adherence (Butler, 1989). Throughout their studies, the competence need or sense of efficacy of college students in PE majors were also enhanced with the increase of professional sports experience and improvement in athletic ability (Wang et al., 2020). By comparison, for college students in non-PE majors, physical activities are usually performed with companions, which is conducive to expanding their network and deepening their emotional connection with their peers (Leversen et al., 2012). Therefore, physical exercise is closely related to the satisfaction of relatedness need. On this basis, this study compared the mediating effects of physical exercise commitment and adherence on life satisfaction on meeting the competence and relatedness needs of college students in PE and non-PE majors. The aim was to reveal the impact of college majors on the relationship between physical exercise and mental health.

In summary, this study examined the effect of physical exercise commitment and adherence on psychological adaption—that is, life satisfaction—in college students. Subsequently, on the basis of the Basic Psychological Need Theory, this study examined the mediating roles of the satisfaction of competence and relatedness needs and their differences among college students in PE and non-PE majors. In addition, given that life satisfaction is readily influenced by individual factors (Li and Lu, 2022), this study intended to include and control for factors such as age, gender, and subjective socioeconomic status of college students in the statistical model. The following hypotheses were thus proposed:

Hypothesis 1: Physical exercise commitment and adherence can positively predict college students’ life satisfaction.
Hypothesis 2: Physical exercise commitment and adherence can positively predict the satisfaction of competence and relatedness needs, which in turn have a positive effect on college students’ life satisfaction.
Hypothesis 3: The satisfaction of competence need has a stronger mediating effect on college students in PE majors than on those in non-PE majors, while the satisfaction of relatedness need has a stronger mediating effect on college students in non-PE majors than on those in PE majors.

## Materials and Methods

### Participants

This study applied the online survey platform, Questionnaire Star, to carry out the survey among college students in Chinese universities. A total of 1,012 valid questionnaires were retrieved. Among the sample, 527 were male and 485 were female; the average age was 19.97 (*SD* = 1.65); and 407 were in PE majors (*M*_age_ = 20.25, *SD*_age_ = 1.65) while 605 were in non-PE majors (*M*_age_ = 19.78, *SD*_age_ = 1.62). Respondents’ subjective evaluations of the socioeconomic status of their families in their city/province and the university they attend were scored on an 11-point scale, and the results were 6.230 ± 1.763 and 6.820 ± 1.669, respectively. Therefore, the respondents in this study were mainly from the middle class. All respondents have signed an informed the consent before filling out the questionnaire and have received payment after completing the study. All content of this study has been approved by the university ethics committee.

### Materials and Procedure

#### Physical Exercise Questionnaire for College Students

The Physical Exercise Questionnaire for College Students was used to measure the physical exercise commitment and adherence of the respondents (Jiang et al., 2018). Physical exercise commitment (e.g., “I have a hard time accepting a lifestyle that lacks physical exercise.”) and adherence (e.g., “I have a habit of exercising.”) each consists of four items. A 5-point scale (1 = *completely disagree*, 5 = *completely agree*) was used, with reverse scores of reverse-coded items, to calculate the mean value of the item. A high score indicates a high level of physical exercise. The Cronbach’s alpha coefficients of physical exercise commitment and adherence were 0.92 and 0.87, respectively.

#### Psychological Needs in Exercise

The Basic Psychological Needs in Exercise Scale (BPNES) was used to measure the respondents’ competence and relatedness needs satisfaction (Vlachopoulos and Michailidou, 2006). Needs satisfaction of competence (e.g., “I feel I have been making a huge progress with respect to the end result I pursue.”) and of relatedness (e.g., “I feel extremely comfortable when with the other exercise participants.”) each includes four items. A 5-point scale (1 = *completely disagree*, 5 = *completely agre*e) was used, and a high score indicates a high level of competence and relatedness needs satisfaction. The Cronbach’s alpha coefficients of both dimensions are above 0.94.

#### Life Satisfaction

This study used the Satisfaction with Life Scale (SWLS) to measure the respondents’ life satisfaction (Diener et al., 1985). This 7-point scale (1 = *completely disagree*, 7 = *completely agree*) consists of five items, such as “The conditions of my life are excellent.” A high score indicates a high level of life satisfaction. In this study, the Cronbach’s alpha coefficient of SWLS was 0.91.

### Data Analysis

This study used questionnaires to measure physical exercise, psychological needs in exercise, and life satisfaction. Descriptive statistics and correlation analysis were performed using SPSS 25.0. With incorporation of the control variables (gender, age, major, and subjective family socioeconomic status), the structural equation model was used in Mplus 8.3 to examine the total effect model (two latent independent variables, one latent dependent variable) and the mediation model (two latent independent variables, two latent mediating variables, and one latent dependent variable). The 95% confidence intervals for the mediating effects were calculated using the bias-corrected bootstrap method (5,000 bootstrap samples). Finally, the difference in the mediation model between college students in PE majors and in non-PE majors was compared using multi-group structural equation modeling. The measurement models of both total and the mediation models were considered good fits that met the standards (total effect model: χ^2^ = 279.905, *df* = 62, RMSEA = 0.059, CFI = 0.979, TLI = 0.973, SRMR = 0.029; mediation model: χ^2^ = 647.034, *df* = 179, RMSEA = 0.051, CFI = 0.978, TLI = 0.974, SRMR = 0.026). The factor loadings |λ| are all greater than 0.5. In addition, the results of the collinearity test showed that the Variance Inflation Factors (VIF) are all no greater than four, indicating no severe collinearity problems (Dormann et al., 2013).

## Results

### Preliminary Analysis

Table 1 presents the mean value, standard deviation, and correlation analysis results of each variable. Both physical exercise commitment (*r* = 0.26, *p* < 0.001) and adherence (*r* = 0.28, *p* < 0.001) were significantly positively correlated with life satisfaction. Physical exercise commitment was significantly positively correlated with the satisfaction of competence needs (*r* = 0.51, *p* < 0.001) and relatedness needs (*r* = 0.48, *p* < 0.001). Physical exercise adherence was significantly positively correlated with the satisfaction of competence need (*r* = 0.63, *p* < 0.001) and relatedness need (*r* = 0.56, *p* < 0.001). Satisfaction of competence need (*r* = 0.37, *p* < 0.001) and relatedness need (*r* = 0.39, *p* < 0.001) were significantly positively correlated with life satisfaction.

**TABLE 1:** The results of descriptive statistics and correlation analysis.

Variables	*M*	*SD*	1	2	3	4	5
(1) Physical exercise commitment	3.45	0.98	1				
(2) Physical exercise adherence	3.32	0.89	0.65***	1			
(3) Competence need satisfaction	3.44	0.92	0.51***	0.63***	1		
(4) Relatedness need satisfaction	3.57	0.94	0.48***	0.56***	0.65***	1	
(5) Life satisfaction	4.51	1.28	0.26***	0.28***	0.37***	0.39***	1

***p < 0.001.

### Mediation Model Analysis

The total effects of physical exercise commitment and adherence on life satisfaction ware tested in a structural equation model. The results showed that the model fitted well (χ2 = 775.103, *df* = 122, RMSEA = 0.073, CFI = 0.940, TLI = 0.929, SRMR = 0.115). The total effect of physical exercise commitment on life satisfaction was marginally significant (β = 0.17, *p* = 0.05) while that of physical exercise adherence on life satisfaction was not significant (β = 0.10, *p* = 0.27).

**FIGURE 1:**
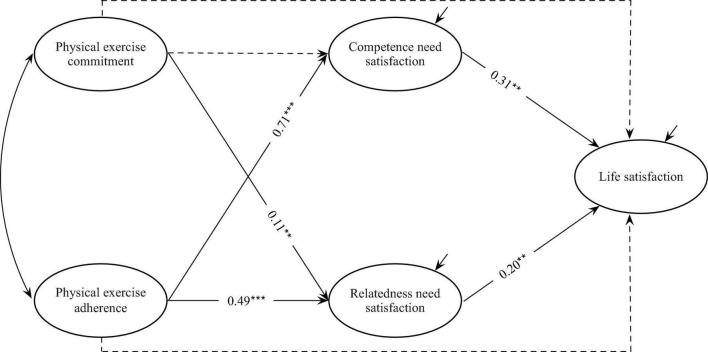
Mediation model from physical exercise to life satisfaction. **p < 0.01, ***p < 0.001; Dashed lines indicate non-significant coefficient.

Competence and relatedness needs satisfaction were incorporated into the total effect model as mediating variables for analysis and the model fitted well (χ^2^ = 1216.310, *df* = 269, RMSEA = 0.059, CFI = 0.956, TLI = 0.948, SRMR = 0.096). Figure 1 showed the results. The effect of physical exercise commitment on competence need satisfaction was not significant (β = 0.04, *p* = 0.67), but could also notably positively predict the relatedness need satisfaction (β = 0.18, *p* < 0.05); and that physical exercise adherence positively predicted the satisfaction of competence needs (β = 0.74, *p* < 0.001) and relatedness needs (β = 0.48, *p* < 0.001). Both competence need satisfaction (β = 0.22, *p* < 0.01) and relatedness need satisfaction (β = 0.14, *p* < 0.01) significantly predicted life satisfaction. The bias-corrected bootstrap method (5,000 bootstrap samples) was used to further test the significance of the mediating effect (see Table 2). The 95% confidence intervals of the mediating effects of both competence and relatedness needs satisfaction on the relationship between physical exercise adherence and life satisfaction did not include 0. The mediation value of competence need satisfaction was moderate whereas that of relatedness need satisfaction was small. By comparison, the 95% confidence interval of the mediating effect of relatedness need satisfaction on the relationship between physical exercise commitment and life satisfaction did not include 0, which indicated a small value, and that of the mediating effect of competence need satisfaction on the relationship between physical exercise commitment and life satisfaction included 0, indicating an insignificant role.

**TABLE 2:** The results of mediation analysis using bootstrap.

Pathways	All participants			Physical education major			Non-physical education major		
			
	Effect	SE	95% CI	Effect	SE	95% CI	Effect	SE	95% CI
PEC → CNS → LS	0.01	0.02	[–0.03, 0.05]	–0.04	0.05	[–0.20, 0.01]	0.02	0.02	[–0.01, 0.067]
PEC → RNS → LS	0.03	0.02	[0.01, 0.07]	0.00	0.10	[–0.03, 0.03]	0.05	0.04	[0.01, 0.12]
PEA → CNS → LS	0.16	0.06	[0.06, 0.26]	0.31	0.13	[0.09, 0.61]	0.09	0.05	[0.01, 0.22]
PEA → RNS → LS	0.07	0.03	[0.02, 0.13]	0.00	0.99	[–0.15, 0.15]	0.04	0.02	[0.01, 0.11]

*PHC, physical exercise commitment, PHA, physical exercise adherence, CNS, competence need satisfaction, RNS, relatedness need satisfaction, LS, life satisfaction.*

### Multigroup Analysis Between Physical Education and Non-physical Education Majors

**FIGURE 2:**
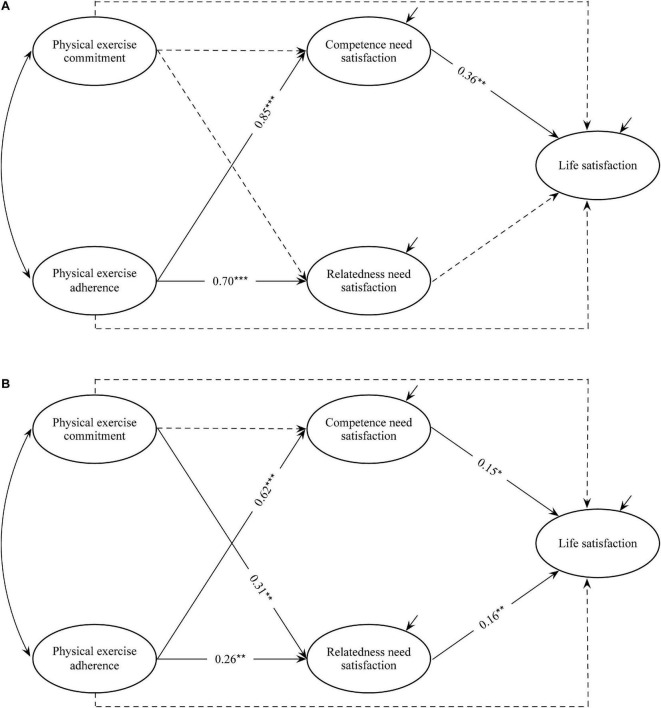
Multi-group mediation model from physical exercise to life satisfaction. *p < 0.05, **p < 0.01, ***p < 0.001; Dashed lines indicate non-significant coefficient. **(A)** Physical education major. **(B)** Nonphysical education major.

First, the above mediation model was tested on the group of college student in PE majors and in non-PE majors to determine whether a multi-group structural equation modeling can be performed (Wang et al., 2011). The results showed that the mediation model was a good fit for two groups (PE majors: χ^2^ = 566.491, *df* = 251, RMSEA = 0.056, CFI = 0.961, TLI = 0.954, SRMR = 0.029; non-PE majors: χ^2^ = 767.396, *df* = 251, RMSEA = 0.059, CFI = 0.955, TLI = 0.947, SRMR = 0.066). Then, under the condition that the loadings and intercepts of the measurement models of the two groups were constrained to be equal, the model with the freely estimated structural path coefficients (model 1) fitted the data well (χ^2^ = 1420.963, *df* = 534, RMSEA = 0.058, CFI = 0.955, TLI = 0.950, SRMR = 0.066). Finally, the model with the structural path coefficients further constrained to be equal (model 2) also fitted the data well (χ^2^ = 1463.251, *df* = 555, RMSEA = 0.057, CFI = 0.954, TLI = 0.951, SRMR = 0.073). The results showed a significant difference between models 1 and 2 (Δχ^2^ = 42.288, Δ*df* = 21, *p* < 0.01), indicating a significant difference in the mediation model between college students in PE and non-PE majors (Figure 2).

Physical exercise commitment had a significant predictive effect on relatedness need satisfaction for the non-PE group (β = 0.307, *p* = 0.001), but not significant for the PE group (β = − 0.03, *p* = 0.75). In addition, this predictive effect on competence need satisfaction was not significant in either the PE or the non-PE group. By comparison, the predictive effect of physical exercise adherence on competence need satisfaction was significant in both PE and non-PE groups (β_*PE*_ = 0.85, *p* < 0.001; β_*non–PE*_ = 0.62, *p* < 0.001). The results of the Wald chi-square test showed that the predictive effect in the PE group was greater than that in the non-PE group (Wald χ^2^ = 4.10, *p* < 0.05). Similarly, the predictive effect of physical exercise adherence on relatedness need satisfaction in the PE group was significantly greater than that in the non-PE group (β_*PE*_ = 0.700, *p* < 0.001; β_*non–PE*_ = 0.263, *p* = 0.004; Wald χ^2^ = 11.972, *p* < 0.001). In both groups, competence need satisfaction had significant predictive effects on life satisfaction (β_*PE*_ = 0.36, *p* < 0.01; β_*non–PE*_ = 0.15, *p* < 0.05). Further comparison of the parameter critical ratios showed that such predictive effects between the two groups had no significant difference (Wald χ^2^ = 2.484, *p* = 0.12). Moreover, the predictive effect of relatedness need satisfaction on life satisfaction was significant in the non-PE group (β = 0.16, *p* < 0.01) but insignificant in the PE group (β = −0.001, *p* = 0.99).

Further bootstrap test (5,000 bootstrap samples) showed that the 95% confidence intervals of the mediating effects of competence need satisfaction on the relationship between physical exercise adherence and life satisfaction of both PE and non-PE groups did not include 0. The mediation value for the former was greater than 0.25, which was considered large, but that for the latter was medium. By comparison, for the non-PE group, the 95% confidence intervals of the mediating effects of relatedness need satisfaction between the relationships of physical exercise commitment/adherence and life satisfaction did not include 0, indicating a small role, while those for the PE group included 0, indicating an insignificant mediating effect.

## Discussion

This study explored the effect and the mechanism of physical exercise commitment and adherence on positive psychological outcomes—that is, life satisfaction—in non-clinical college students, revealing that physical exercise could not only alleviate psychological symptoms but also improve individual well-being and achieve the ultimate goal of staying mentally healthy, which extended the empirical research on promoting mental health through physical exercise. At the same time, this study compared the differences between college students in PE majors and non-PE majors, which deepened the understanding of the relationship between physical exercise and mental health. On this basis, this study put forward practical suggestions. The major findings were as follows: (1) Physical exercise commitment positively predicted college students’ satisfaction with life; (2) Physical exercise commitment only positively predicted college students’ satisfaction with life through relatedness need satisfaction, while physical exercise adherence did the same through both competence and relatedness needs satisfaction; (3) The role of competence need satisfaction was emphasized in the relationship between physical exercise and the life satisfaction of college students majoring in PE.

Consistent with Hypothesis 1, physical exercise commitment positively predicted the subjective well-being of college students. However, after incorporating the effect of physical exercise commitment, physical exercise adherence failed to positively predict the subjective well-being of college students, which was inconsistent with the current hypothesis and the results of previous studies. Although direct examinations of the effects of physical exercise commitment and adherence on mental health were rare, empirical studies on their respective effects have found that these two factors have significant effects on mental health (Pini et al., 2007; Sullins, 2019). The present findings suggested that, first, future research needs to incorporate both physical exercise commitment and adherence to clarify their respective effects. Second, this study also highlighted the role of physical exercise commitment in college students’ satisfaction with life. Specifically, commitment is a motivational component of physical exercise, while adherence is a behavioral component (Gomes and Capelão, 2013; Chapman et al., 2015). In other words, the mental states of college students have a more pronounced effect on life satisfaction than their behaviors. Notably, although the total effect of physical exercise adherence on college students’ satisfaction with life was not significant, its indirect effect has been proven by the subsequent mediating effect analysis, especially for the group of college students majoring in PE.

This study found that competence and relatedness needs satisfaction had a mediating effect on the relationship between physical exercise and college students’ satisfaction with life. This finding supported the Basic Psychological Need Theory (Rodriguez, 2020) and verified Hypothesis 2, that was, physical exercise effectively improved college students’ sense of competence and self-efficacy. In addition, physical exercise satisfied their relatedness need through cooperation and interaction with others, thereby improving their life satisfaction and enhancing their psychological resilience. At the same time, these findings brought a unique perspective to the understanding of the difference between physical exercise commitment and adherence; the former had an impact on college students’ satisfaction with life only through the mediating effect of relatedness need satisfaction, while the latter affected college students’ satisfaction with life through both relatedness and competence needs satisfaction. Physical exercise adherence is a manifestation of behavior, which requires effort of will (Chapman et al., 2015), and the successful implementation of behaviors can satisfy the competence need of college students. Meanwhile, physical exercise is also an important medium for interpersonal communication, enabling individuals to connect with others and be accepted, thereby satisfying their relatedness need (Doré et al., 2020). The satisfaction of competence and relatedness needs allowed individuals to have a positive self-recognition and evaluation, gained a sense of belonging, and thus improved life satisfaction (González et al., 2016). However, physical exercise commitment is a planned behavior and motivational state (Gomes and Capelão, 2013), and hardly reflected individuals’ advantage in their ability, which explained the insignificant mediating effect of competence need satisfaction.

Notably, a significant difference was observed in the mediating effects of competence and relatedness needs satisfaction between college students in PE and non-PE majors, reflecting the particularity of the former. The results showed that among college students majoring in PE, only competence need satisfaction had a mediating effect on the relationship between physical exercise adherence and their satisfaction with life. While physical exercise may be an important medium for interpersonal communication for college students not majoring in PE, it was important for training and professional competence improvement for college students majoring in PE, and therefore competence need satisfaction played a vital role. This finding was consistent with previous studies that had also found high scores of an individual’s competence need satisfaction in more professional and organized sports activities (Fraguela-Vale et al., 2020). In addition, only physical exercise adherence had an impact on the satisfaction with life among college students majoring in PE, but both physical exercise commitment and adherence affected life satisfaction among college students not majoring in PE. One possible reason was that the physical exercise commitment that remained as a motivation but never converted to action cannot satisfy the competence need of college students majoring in PE, and thus it failed to exert a positive impact on life satisfaction.

## Implications and Limitations

The practical implications of this study were as follows. First, this study proved the importance of physical exercise in improving college students’ satisfaction with life, which was conducive to suggesting relevant practical strategies to achieve the ultimate goal of mental health, that was, improving individual well-being. Second, this study explained the relationship between physical exercise and college students’ satisfaction with life based on the Basic Psychological Need Theory. The mediating roles of competence and relatedness needs satisfaction were revealed, thereby providing theoretical and empirical foundations for an in-depth understanding of this relationship. The implications were that future physical exercise intervention programs can improve college students’ satisfaction with life by meeting their competence need and relatedness needs during physical exercise. Finally, this study revealed the difference in the mediating effects of competence and relatedness needs satisfaction between college students in PE majors and non-PE majors. The unique role of competence need satisfaction among college students majoring in PE was emphasized, and was conducive to improving the pertinence and effectiveness of relevant intervention practice strategies.

Despite its certain contributions to the understanding of the relationship between physical exercise and college students’ satisfaction with life and to designing activities in practice, this study did have the following limitations. First, given its cross-sectional design, this study failed to accurately describe the causal relationship between physical exercise and college students’ satisfaction with life, and required follow-up studies or randomized controlled trials for further verification. Second, this study focused solely on college students, which led to the difficulties of generalizing the findings to other groups. Third, in terms of individual positive psychological outcomes, this study only examined the life satisfaction of college students, which was inadequate to provide a wholistic perspective of how physical exercise increased individual psychological adaption, and more supporting evidence from other perspectives were necessary. Finally, this study discussed the psychological mechanism of the effect of physical exercise on college students’ satisfaction with life but excluded its physiological mechanism. Future research needs to integrate physiological and psychological mechanisms to construct an explanatory chain of how physical exercise affects college students’ mental health.

## Data Availability Statement

The raw data supporting the conclusions of this article will be made available by the authors, without undue reservation.

## Ethics Statement

The studies involving human participants were reviewed and approved by the Research Ethical Committee of Hunan Normal University. The patients/participants provided their written informed consent to participate in this study.

## Author Contributions

YZ was involved in the conceptualization, data collection, data analysis, interpretation, and wrote the manuscript. MR was involved in the conceptualization and interpretation. SZ was involved in the conceptualization, ethical approval, and interpretation. All authors read and approved the final manuscript.

## Conflict of Interest

The authors declare that the research was conducted in the absence of any commercial or financial relationships that could be construed as a potential conflict of interest.

## Publisher’s Note

All claims expressed in this article are solely those of the authors and do not necessarily represent those of their affiliated organizations, or those of the publisher, the editors and the reviewers. Any product that may be evaluated in this article, or claim that may be made by its manufacturer, is not guaranteed or endorsed by the publisher.
